# Dual Targeting of FGFR4 and PI3K–mTOR Suppresses Tumor-Associated Phenotypes in Pancreatic Ductal Adenocarcinoma

**DOI:** 10.3390/ijms27177722

**Published:** 2026-08-28

**Authors:** Joseph A. Goode, Savannah A. Harris, Deborah A. Altomare

**Affiliations:** Burnett School of Biomedical Sciences, College of Medicine, University of Central Florida, Orlando, FL 32827, USA; joseph.goode@ucf.edu (J.A.G.); savannah.harris@ucf.edu (S.A.H.)

**Keywords:** pancreatic cancer, mechanistic target of rapamycin (mTOR), phosphoinositide 3-kinases (PI3K), fibroblast growth factor 19 (FGF19), fibroblast growth factor receptor 4 (FGFR4), combination therapy

## Abstract

Pancreatic ductal adenocarcinoma (PDAC) is characterized by extensive adaptive signaling plasticity and metabolic reprogramming that contribute to therapeutic resistance. While the PI3K–mTOR pathway is a central regulator of these processes, the role of the FGF19–FGFR4 axis and its interaction with PI3K-mTOR signaling remains incompletely defined in PDAC. This study investigated whether co-targeting FGFR4 and PI3K-mTOR signaling could overcome adaptive pathway reactivation and suppress tumor-promoting phenotypes. PDAC cell lines representing a spectrum of FGFR4 dependence were treated with the selective FGFR4 inhibitor fisogatinib in combination with the clinically relevant PI3K–mTOR inhibitor gedatolisib. Transcriptomic analyses of TCGA data, along with molecular and functional responses, were evaluated. Transcriptomic analysis demonstrated a positive association between FGFR4 and PI3K–mTOR signaling and linked combined pathway components with poorer overall survival. Across heterogeneous PDAC models, combined inhibition reduced viability, clonogenic survival, migration, and cell-cycle progression more consistently than monotherapy. Apoptosis induction was driven principally by fisogatinib and combination treatment, resulting in comparable apoptotic responses across cell lines. Mechanistically, combined inhibition converged on increased 4E-BP1 inhibitory activity, despite compensatory ERK-RSK pathway activation, indicating that adaptive MAPK was insufficient to restore downstream translational output. In FGFR4-dependent cells, the combined inhibition also reduced secretion of the FGFR4 ligand FGF19. The FGFR4 and PI3K–mTOR signaling comprises a partially interconnected network in PDAC that converges on 4E-BP1-dependent translational control. Dual pathway inhibition consistently and additively suppressed tumor-associated phenotypes despite compensatory MAPK activation, with the clearest added benefit over fisogatinib alone in migration, cell-cycle control, and 4E-BP1 modulation, supporting translational regulation as a shared therapeutic vulnerability. These findings provide a preliminary rationale for biomarker-guided strategies targeting FGFR4 and PI3K–mTOR signaling in pancreatic cancer.

## 1. Introduction

Pancreatic ductal adenocarcinoma (PDAC) is the third leading cause of cancer-related mortality in the United States despite being only the tenth most common, largely due to late diagnosis, aggressive tumor biology, and resistance to available therapies [1,2,3,4]. Oncogenic KRAS-driven signaling and extensive pathway redundancy contribute to therapeutic failure, underscoring the need for rational combination strategies that target compensatory survival mechanisms.

The fibroblast growth factor 19 (FGF19)–fibroblast growth factor receptor 4 (FGFR4) signaling axis has emerged as a potential therapeutic target in multiple cancers. FGFR4, a transmembrane receptor tyrosine kinase involved in hepatocyte metabolism and bile acid homeostasis, can promote tumor cell proliferation, survival, and motility when aberrantly activated [5,6,7,8,9,10,11,12]. Upregulation of the FGF19-FGFR4 axis has been implicated in gastric cancers and pharmacologic inhibition of FGFR4 using selective agents such as fisogatinib (BLU-554) and BLU9931 has demonstrated clinical feasibility in FGF19-positive hepatocellular carcinoma and esophageal squamous cell carcinoma, respectively [13,14,15,16]. However, in PDAC, FGFR4 inhibition alone has shown variable and sometimes paradoxical effects on tumor growth and metastatic behavior, suggesting the presence of compensatory signaling mechanisms that limit its efficacy [8,9,17].

One such mechanism involves activation of downstream survival pathways, including PI3K-AKT and mechanistic target of rapamycin (mTOR) signaling. mTOR is a central regulator of cell growth and metabolism and has been widely investigated as a therapeutic target in PDAC. However, clinical studies of mTOR inhibitors have demonstrated limited benefits, in part due to feedback activation of upstream receptor tyrosine kinases and incomplete pathway suppression [18,19,20,21,22]. Inhibition of mTORC1 alone can relieve negative feedback on insulin receptor substrate 1 (IRS1), leading to reactivation of PI3K-AKT and MAPK signaling, while continued mTORC2 activity further sustains AKT signaling [20,23,24,25]. These responses have also been seen across other GI cancers, including colorectal and esophageal [26,27]. These adaptive responses highlight the limitations of single-agent targeting and support the need for strategies that more comprehensively suppress pathway reactivation.

Dual inhibition of PI3K and mTOR represents one such approach. Gedatolisib is a dual PI3K-mTOR inhibitor that targets both mTORC1 and mTORC2, thereby limiting feedback-driven reactivation of downstream signaling pathways observed with selective mTORC1 inhibitors such as rapalogs [24,25,28,29]. Given the interplay between FGFR4 signaling and PI3K-AKT-mTOR pathway activation, combined targeting of these pathways may provide a more effective therapeutic strategy.

In this study, we investigated the functional relationship between FGFR4 and PI3K-mTOR signaling in PDAC and tested whether combined pathway inhibition could overcome adaptive signaling responses that limit anti-tumor efficacy of single-agent therapies. Using selective inhibition of FGFR4 with fisogatinib and PI3K-mTOR with gedatolisib, we evaluated the effects of co-targeting these pathways on tumor-associated phenotypes and downstream signaling networks. Our findings showed 4E-BP1-dependent translational control as a point of convergence between FGFR4 and PI3K-mTOR signaling and support biomarker-guided therapeutic strategies targeting these signaling axes in PDAC.

## 2. Results

### 2.1. Transcriptomic Analysis Suggests Limited Co-Variation Between FGFR4 and PI3K–mTOR Signaling in PDAC

To investigate the relationship between FGFR4 and PI3K–mTOR signaling in pancreatic ductal adenocarcinoma (PDAC), transcriptomic data from the TCGA-PAAD cohort (n = 182) were analyzed using composite pathway activity scores derived from canonical pathway components. Correlation analysis demonstrated a modest but statistically significant positive association between FGFR4-axis (*FGFR4* + *FGF19*) and PI3K–mTOR-axis (*PIK3CA*, *MTOR*, *PDK1*) (Spearman ρ = 0.178, *p* = 0.016), indicating partial coordination of these signaling programs across tumors while also highlighting substantial inter-tumoral heterogeneity. Patients were next stratified into four groups based on median FGFR4 and PI3K–mTOR pathway expression: Low FGFR4/Low MTOR, Low FGFR4/High MTOR, High FGFR4/Low MTOR, and High FGFR4/High MTOR axes. Kaplan–Meier analysis demonstrated heterogeneous overall survival across these groups, although the overall four-group comparison did not reach statistical significance (log-rank χ^2^ = 5.66, *p* = 0.129).

**Figure 1:**
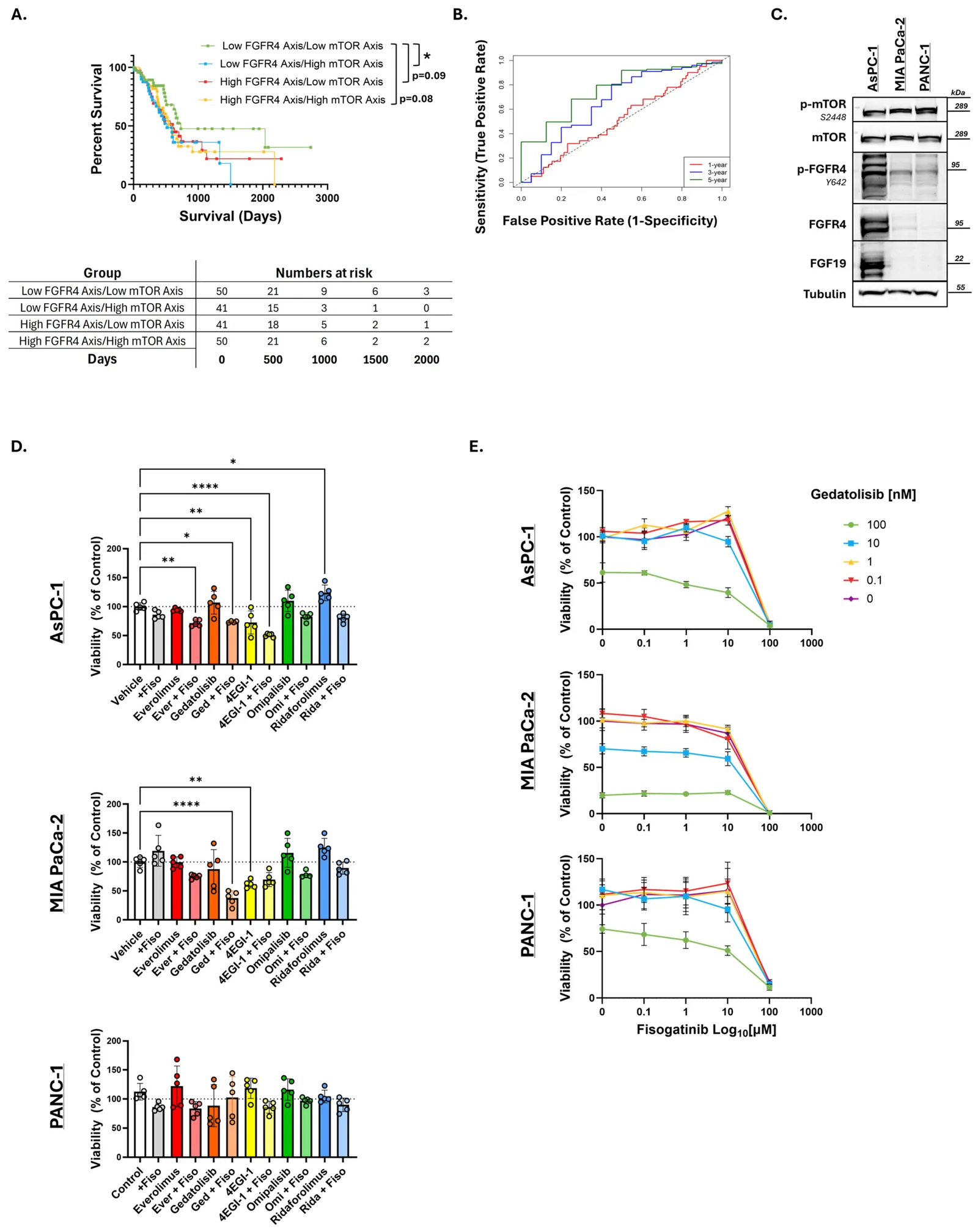
FGFR4 and PI3K–mTOR signaling are associated in PDAC and support evaluation of combined pathway inhibition. (**A**) Kaplan–Meier curves of overall survival (OS) in the TCGA-PAAD cohort stratified into four groups based on median FGFR4 and PI3K–mTOR axis expression (Low FGFR/Low mTOR, Low FGFR/High mTOR, High FGFR/Low mTOR, High FGFR/High mTOR axes; n = 182, TCGA-PAAD cohort), with the number of patients at risk in each group shown below the plot at 500-day intervals. Survival differences were assessed using the log-rank test with pairwise comparisons performed relative to the Low FGFR/Low mTOR reference group. Patients with activation of either or both signaling axes exhibited poorer survival compared with the dual-low reference group. (**B**) Time-dependent ROC analysis demonstrating predictive prognostic performance of the combined FGFR4 and PI3K–mTOR signature at 1, 3, and 5 years. AUC values are indicated. (**C**) Immunoblot analysis comparing relative baseline expression of FGF19–FGFR4 and mTOR pathway components across cell lines loaded at 50 µg total protein on the same blot. AsPC-1 cells exhibited elevated FGF19–FGFR4 axis activity, whereas MIA PaCa-2 and PANC-1 represented lower FGFR4-axis contexts. mTOR pathway expression was detected across all models. (**D**) Targeted inhibitor screen of cell viability following treatment with mTOR pathway inhibitors (everolimus, 1 µM; gedatolisib, 10 nM; 4EGI-1, 10 µM; omipalisib, 10 nM; ridaforolimus, 10 nM) ± fisogatinib (10 µM) for 72 h across PDAC cell lines. Gedatolisib demonstrated the most consistent combinatorial activity across responsive models. (**E**) Dose–response analysis to identify optimal working concentrations of gedatolisib and fisogatinib following 72 h treatment. Data in (**D**,**E**) represent mean ± SD from n = 5. Significance was calculated using one-way ANOVA with multiple comparisons. Statistics: * *p* < 0.05, ** *p* < 0.01, **** *p* < 0.0001.

Pairwise comparisons relative to the Low FGFR4/Low MTOR axes reference group revealed a consistent pattern of reduced survival across multiple pathway-activated states. The Low FGFR4/High MTOR axes subgroup exhibited significantly decreased overall survival (*p* = 0.019). Similarly, both High FGFR4/Low MTOR and High FGFR/High MTOR axes groups demonstrated trends toward poorer survival (*p* = 0.089 and *p* = 0.077, respectively), although these did not reach statistical significance (Figure 1A). Collectively, these findings indicate that activation of either PI3K–mTOR or FGFR4-associated signaling, alone or in combination, is associated with adverse survival patterns relative to the dual-low reference group. The strongest effect was observed in the MTOR-high/FGFR4-low context, while FGFR4-associated groups showed consistent but more modest trends, supporting a model in which both pathways contribute to poor prognosis in a context-dependent manner in PDAC.

Time-dependent receiver operating characteristic (ROC) analysis was performed to evaluate the prognostic performance of the combined FGFR4 and PI3K–mTOR pathway model. Discriminatory ability was limited at 1 year (AUC = 0.52) but improved at later time points, reaching AUCs of 0.67 at 3 years and 0.76 at 5 years (Figure 1B). Given the modest correlation between FGFR4 and PI3K–mTOR axis activity (ρ = 0.178), and the non-significant omnibus log-rank comparison (*p* = 0.129), these transcriptomic findings are best interpreted as hypothesis-generating. Discriminatory performance improved at later time points (3- and 5-year AUC of 0.67 and 0.76, respectively) relative to 1 year (AUC = 0.52), suggesting that cumulative pathway effects on survival may become more apparent over longer follow-up. Together, these findings provide rationale for directly testing the functional relationship between FGFR4 and PI3K–mTOR signaling using pharmacologic inhibition in PDAC cell models, as described below.

### 2.2. PDAC Models with Distinct FGFR4-Axis Activity Exhibit Differential Sensitivity to Combined FGFR4 and PI3K–mTOR Inhibition

To evaluate whether response to FGFR4 and PI3K-mTOR co-targeting varies according to baseline pathway activity, PDAC cell lines representing distinct FGFR4 signaling states were examined. AsPC-1, MIA PaCa-2, and PANC-1 cells represent distinct signaling profiles with reported differences in growth factor dependence and sensitivity to PI3K-mTOR pathway inhibition [30,31,32,33]. AsPC-1 cells exhibited elevated FGF19-FGFR4 axis activity, with high expression of FGF19, FGFR4, and phosphorylated FGFR4 (Figure 1C and Figure S2A). MIA PaCa-2 and PANC-1 cells in this comparison displayed lower FGFR4-axis activity. Expression of mTOR and phosphorylated mTOR was broadly comparable across all cell lines (Figure 1C and Figure S2A). These findings established a panel encompassing both FGFR4-high and FGFR4-low signaling contexts for subsequent evaluation of pathway co-targeting.

A targeted inhibitor screen was performed to identify mTOR-pathway-directed pharmaceutical agents that enhance the effects of the FGFR4 inhibitor fisogatinib (Figure 1D). In AsPC-1 cells, single-agent fisogatinib or mTOR inhibitors alone resulted in minimal effects on viability, whereas several combinations significantly reduced viability more effectively than either monotherapy. MIA PaCa-2 cells showed a significant reduction in viability when treated specifically with a combination of fisogatinib and gedatolisib. In contrast, PANC-1 cells were largely refractory to both single-agent and combination treatments and demonstrated a different sensitivity to this combination than MIA PaCa-2. These findings indicate that sensitivity to FGFR4 and PI3K-mTOR co-targeting varies across PDAC models and is not predicted solely by baseline FGFR4 abundance.

The metastatic PDAC cell line L3.6pl also demonstrated sensitivity in the targeted inhibitor screen, inclusive of combined fisogatinib and gedatolisib treatment (Figure S1A), although this cell line was excluded from subsequent mechanistic analyses due to potential bias from its derivation from repeated passaging in animals [34]. We then performed ZIP drug interaction analysis with AsPC-1, MIA PaCa-2, and PANC-1 as an exploratory assessment of combination effects (Figure S1B). The combination reduced cell viability in responsive models and suggested that the observed effects were largely additive (Figure S1C).

Based on the mTOR inhibitor screening results, gedatolisib was selected for its combined viability reduction in combination with fisogatinib and its potential clinical translatability as the mTOR targeting strategy due to its combined inhibition of PI3K and both mTOR complexes, thereby limiting feedback reactivation associated with selective mTORC1 inhibition. This selection was further supported by superior combinatorial activity with fisogatinib and its more advanced clinical development relative to earlier-generation PI3K-mTOR inhibitors such as omipalisib. Importantly, the concentration ranges were informed by reported clinical exposure ranges and were chosen to achieve consistent pathway modulation while preserving measurable cell viability for downstream assays [13,29,35]. This dosing strategy avoids supraphysiologic exposure conditions that may introduce non-specific or off-target cytotoxic effects, while maintaining biologically effective pathway inhibition in vitro.

Dose–response analysis identified 10 nM gedatolisib and 10 μM fisogatinib as the optimal working concentrations for subsequent experiments (Figure 1E). These doses resulted in measurable growth inhibition while preserving sufficient viability for downstream functional assays. Notably, lower fisogatinib concentrations combined with low-dose gedatolisib produced paradoxical increases in proliferation in MIA PaCa-2 cells, consistent with adaptive signaling responses to partial pathway inhibition [8,9,17]. Collectively, these findings demonstrate heterogeneous dependence on FGFR4 and PI3K-mTOR signaling across PDAC models and identify gedatolisib as the most suitable partner for subsequent dual-targeting studies.

### 2.3. Fisogatinib Preferentially Limits Long-Term Clonogenic Expansion

**Figure 2:**
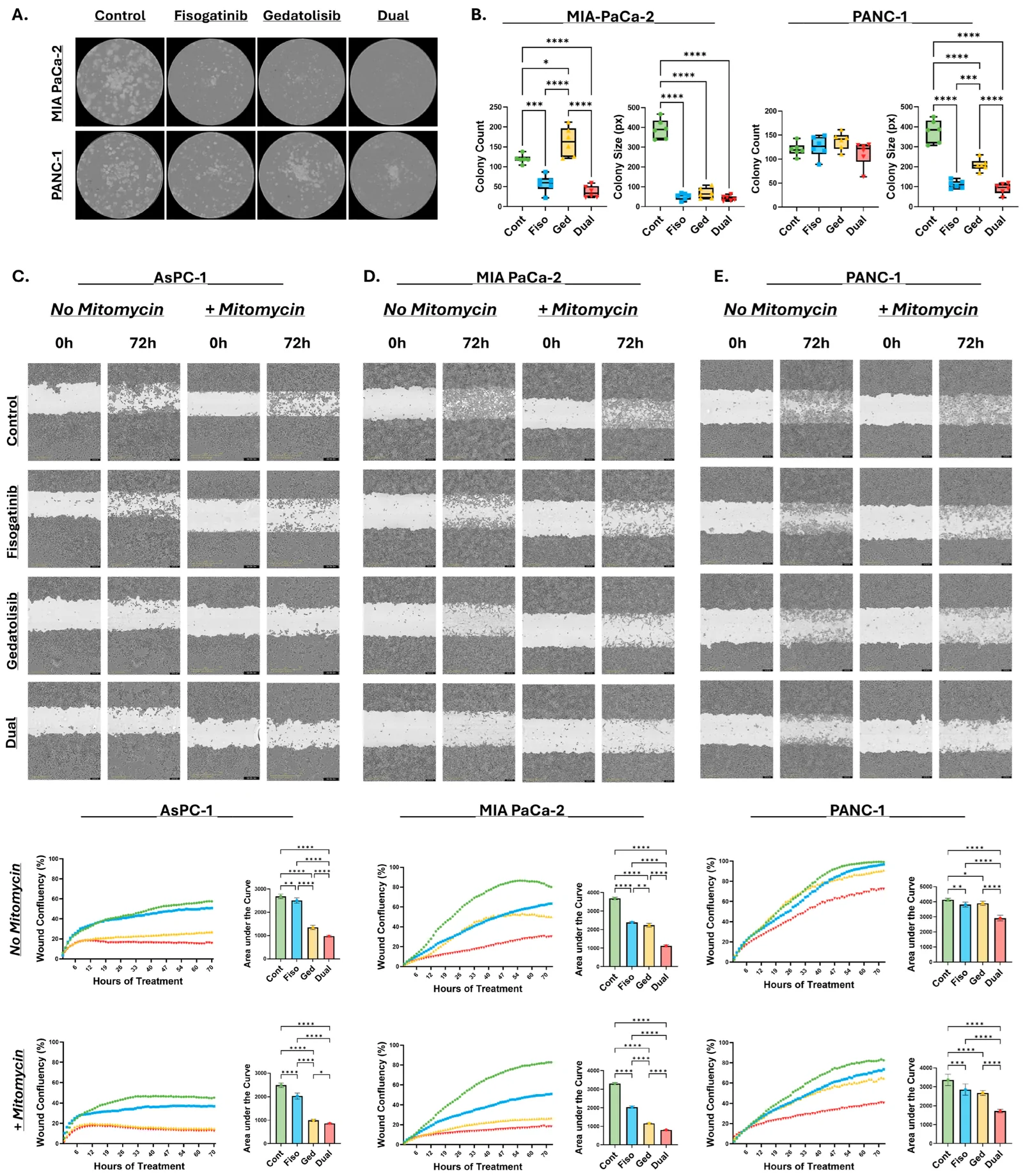
FGFR4 and PI3K-mTOR inhibition suppresses clonogenic growth and migration in PDAC models. (**A**) Representative clonogenic assays of MIA PaCa-2 and PANC-1 cells following treatment with vehicle, fisogatinib (10 µM), gedatolisib (10 nM), or combination for 9 days with treatment replenishment every 3 days. (**B**) Quantification of colony number and colony size (n = 6). Fisogatinib-containing treatments reduced clonogenic growth, with the most consistent effects observed on colony expansion. (**C**–**E**) Scratch-wound assays evaluating migration in (**C**) AsPC-1, (**D**) MIA PaCa-2, and (**E**) PANC-1 cells treated with vehicle, fisogatinib (10 µM), gedatolisib (10 nM), or combination and monitored over 72 h. Where indicated, cells were pretreated with or without mitomycin C (10 µg/mL, 2 h) to inhibit proliferation-dependent wound closure. Scale bars represent 600 µm for AsPC-1 + mitomycin and 400 µm for the remaining images. Relative wound density is shown over time with graphical data represented by AUC ± SD of the wound healing curves (n = 8) corresponding to curves of the same color. Dual inhibition produced the greatest reduction in migratory capacity across models, with inhibitory effects partially retained in the presence of mitomycin C. Significance was calculated using one-way ANOVA for (**B**) and two-way ANOVA for (**C**–**E**). Statistics: * *p* < 0.05, ** *p* < 0.01, *** *p* < 0.001, **** *p* < 0.0001.

Fisogatinib was the principal driver of clonogenic suppression across models, with combination treatment providing minimal additional benefit beyond fisogatinib alone, consistent with the largely additive interaction observed by ZIP analysis (Figure S1B). To evaluate long-term clonogenic survival, colony formation assays were performed over 9 days, with media and drug replenishment every 3 days. AsPC-1 cells were excluded because they failed to form discrete colonies and instead grew as a monolayer, precluding reliable quantification. Subsequent analysis therefore focused on MIA PaCa-2 and PANC-1 cells (Figure 2A,B). In MIA PaCa-2 cells, fisogatinib reduced colony number and size. In contrast, gedatolisib increased colony number while reducing colony size, indicating preservation of colony initiation but impaired subsequent colony expansion. Combined inhibition reduced colony number and size relative to vehicle and gedatolisib but did not further suppress clonogenic growth beyond fisogatinib alone. PANC-1 cells preserved colony initiation regardless of treatment, as assessed by the absence of significant changes in colony count, but reduced colony size. Dual treatment reduced colony size relative to gedatolisib but did not further reduce colony expansion beyond fisogatinib alone. Overall, clonogenic responses varied between models. However, fisogatinib-containing treatments consistently produced the strongest effect on reduced colony growth, primarily through suppression of colony expansion. Combination treatment did not substantially improve these effects beyond fisogatinib alone.

### 2.4. Combined FGFR4 and PI3K–mTOR Targeting Limits Migratory Capacity in PDAC Cells

To assess the effects of FGFR4 and PI3K-mTOR co-inhibition on PDAC cell migration, real-time scratch-wound assays were performed over 72 h in the presence or absence of mitomycin C to distinguish migratory responses from proliferation-dependent wound closure. Across all three cell lines, fisogatinib plus gedatolisib significantly impaired wound closure compared to vehicle and single agents (Figure 2C–E). In AsPC-1 cells, monotherapies modestly delayed wound closure, but the combination produced the greatest inhibition, as assessed by reduced relative wound density. This effect was attenuated but remained detectable with mitomycin C, indicating partial proliferation-independent activity (Figure 2C). MIA PaCa-2 cells were more responsive to gedatolisib than fisogatinib monotherapy, while combination treatment resulted in the greatest reduction in migration. These differences were attenuated in the presence of mitomycin C, suggesting a contribution of proliferation-dependent mechanisms (Figure 2D). PANC-1 cells also exhibited significant reductions in wound closure following combination treatment, and this effect persisted in the presence of mitomycin C (Figure 2E).

While monotherapies moderately delayed wound closure in a cell line-dependent manner, combined inhibition resulted in the greatest suppression of migration across all three PDAC models. Inhibitory effects remained detectable in the presence of mitomycin C, indicating that reduced migration was not solely attributable to impaired proliferation. The parallel mitomycin C condition was included specifically to isolate migration from proliferation-dependent wound closure, and the retention of a combination effect under mitomycin C treatment supports a genuine anti-migratory effect rather than an artifact of serum-starvation-driven growth arrest. Collectively, these findings demonstrate that combined FGFR4 and PI3K-mTOR targeting limits migratory capacity across heterogeneous PDAC models.

### 2.5. Fisogatinib-Containing Treatments Are Associated with Apoptotic Responses in PDAC Cells

**Figure 3:**
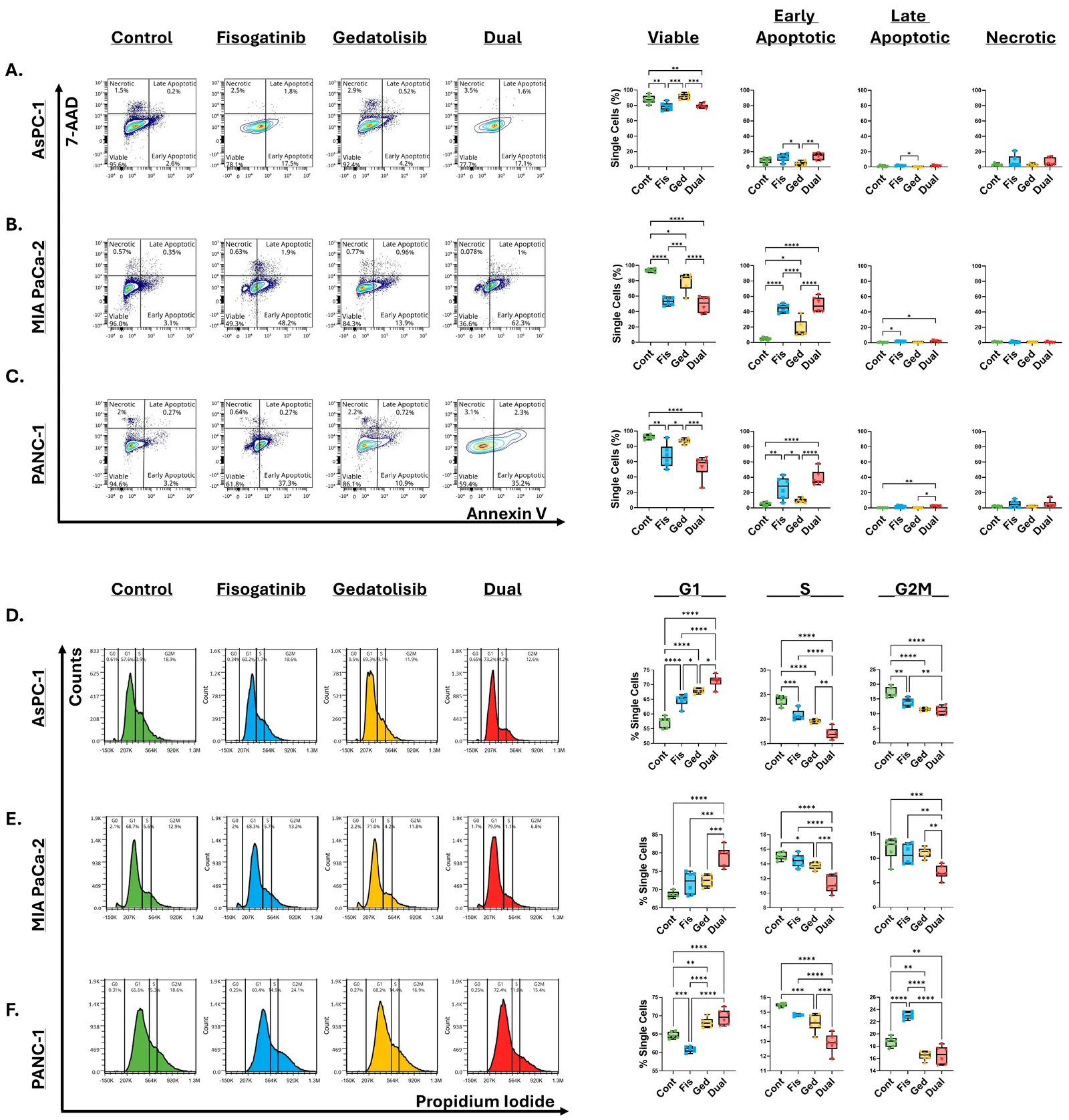
Fisogatinib-containing treatments are associated with increased apoptosis, and dual pathway inhibition promotes cell-cycle arrest in PDAC cells. (**A**–**C**) Annexin V/7-AAD flow cytometric analysis of apoptosis in (**A**) AsPC-1, (**B**) MIA PaCa-2, and (**C**) PANC-1 cells following 48 h treatment with vehicle, fisogatinib (10 µM), gedatolisib (10 nM), or combination. Representative quadrant plots and quantification of viable, early apoptotic, late apoptotic, and necrotic populations are shown. Across all three models, fisogatinib-containing treatments produce the largest apoptotic responses. (**D**–**F**) Propidium iodide cell-cycle analysis of fixed cells following 24 h treatment in (**D**) AsPC-1, (**E**) MIA PaCa-2, and (**F**) PANC-1 cells showing distribution across G1, S, and G2/M phases. Combination treatment altered cell-cycle progression and promoted accumulation of cells in G1 across PDAC models. Data represents mean ± SD from n = 6. Significance was calculated using one-way ANOVA with multiple comparisons. Statistics: * *p* < 0.05, ** *p* < 0.01, *** *p* < 0.001, **** *p* < 0.0001.

Fisogatinib was the principal driver of apoptotic responses across models, with combination treatment producing comparable but not significantly greater effects, which is also consistent with the additive pattern observed by ZIP analysis (Figure S1B). Given the reductions in viability, clonogenic growth, and migration observed following combined FGFR4 and PI3K-mTOR inhibition, we next assessed whether these effects reflected cell death, altered cell cycle progression, or both. Across all three PDAC cell models, no significant increase in necrotic (7-AAD+/Annexin V−) cell populations were observed relative to vehicle controls following 48 h of treatment, indicating that treatment-associated growth suppression was not attributable to nonspecific cytotoxic injury (Figure 3A–C). Instead, apoptosis represented the predominant form of cell death and was most evident in fisogatinib-containing treatment groups. AsPC-1 cells exhibited modest but significant reductions in viability and increases in early apoptotic populations following fisogatinib treatment, whereas gedatolisib alone exhibited minimal effects relative to vehicle controls. Combination treatment resulted in similarly reduced viability and increased apoptotic response, although the effect was not significantly different from fisogatinib alone (Figure 3A). MIA PaCa-2 cells exhibited the greatest overall apoptotic response. Fisogatinib, gedatolisib, and combination treatment all significantly increased early apoptosis relative to vehicle controls. Notably, gedatolisib alone significantly increased apoptosis in MIA PaCa-2 cells, although the magnitude of this response was not greater than that observed with fisogatinib. No statistically significant difference was observed between fisogatinib and the combination treatment groups (Figure 3B). Similar to AsPC-1 cells, fisogatinib and combination reduced viability and increased apoptotic populations in PANC-1 cells, whereas gedatolisib alone had minimal effects. No statistically significant difference was observed between fisogatinib alone and the combination treatment groups (Figure 3C). Collectively, these findings indicate that apoptosis, rather than necrosis, was the predominant mode of treatment-associated cell death in these PDAC models. Fisogatinib-containing treatments were associated with the largest apoptotic responses, whereas combined FGFR4 and PI3K-mTOR inhibition did not significantly increase apoptotic populations beyond that of fisogatinib alone.

### 2.6. Combined FGFR4 and PI3K-mTOR Inhibition Promotes G1 Accumulation in PDAC Cells

To determine whether reduced proliferation was associated with altered cell cycle progression, cells were treated for 24 h and analyzed by flow cytometry after fixation and propidium iodide staining. In AsPC-1 cells, both fisogatinib and gedatolisib increased the G1 phase fraction and reduced S phase, whereas combination treatment resulted in the most pronounced G1 accumulation and reduction in S and G2/M phases (Figure 3D). MIA PaCa-2 cells showed a similar but more graded response (Figure 3E). Fisogatinib modestly increased G1, whereas gedatolisib more clearly promoted G1 accumulation and reduced S phase entry. Combination treatment further increased G1 and reduced both S and G2/M populations relative to either monotherapy. In PANC-1 cells, fisogatinib alone reduced G1 and increased G2/M populations (Figure 3F). Gedatolisib increased G1 and reduced S and G2/M, while combination treatment restored and enhanced a G1-dominant profile.

Collectively, these findings demonstrate that combined FGFR4 and PI3K-mTOR inhibition alters cell cycle progression across PDAC models and results in the most consistent accumulation of cells in G1, with the exception of fisogatinib monotherapy in PANC-1 cells, which instead reduced G1 and increased G2/M. These effects parallel the reductions in proliferation, clonogenic growth, and migration observed following dual pathway inhibition. As cells were serum-starved prior to treatment, serum withdrawal itself may have contributed to baseline G1 arrest independent of drug treatment, a factor to consider when interpreting the magnitude of treatment-associated cell-cycle effects.

### 2.7. Combined FGFR4 and PI3K-mTOR Inhibition Is Associated with Increased 4E-BP1 Suppressive Activity

**Figure 4:**
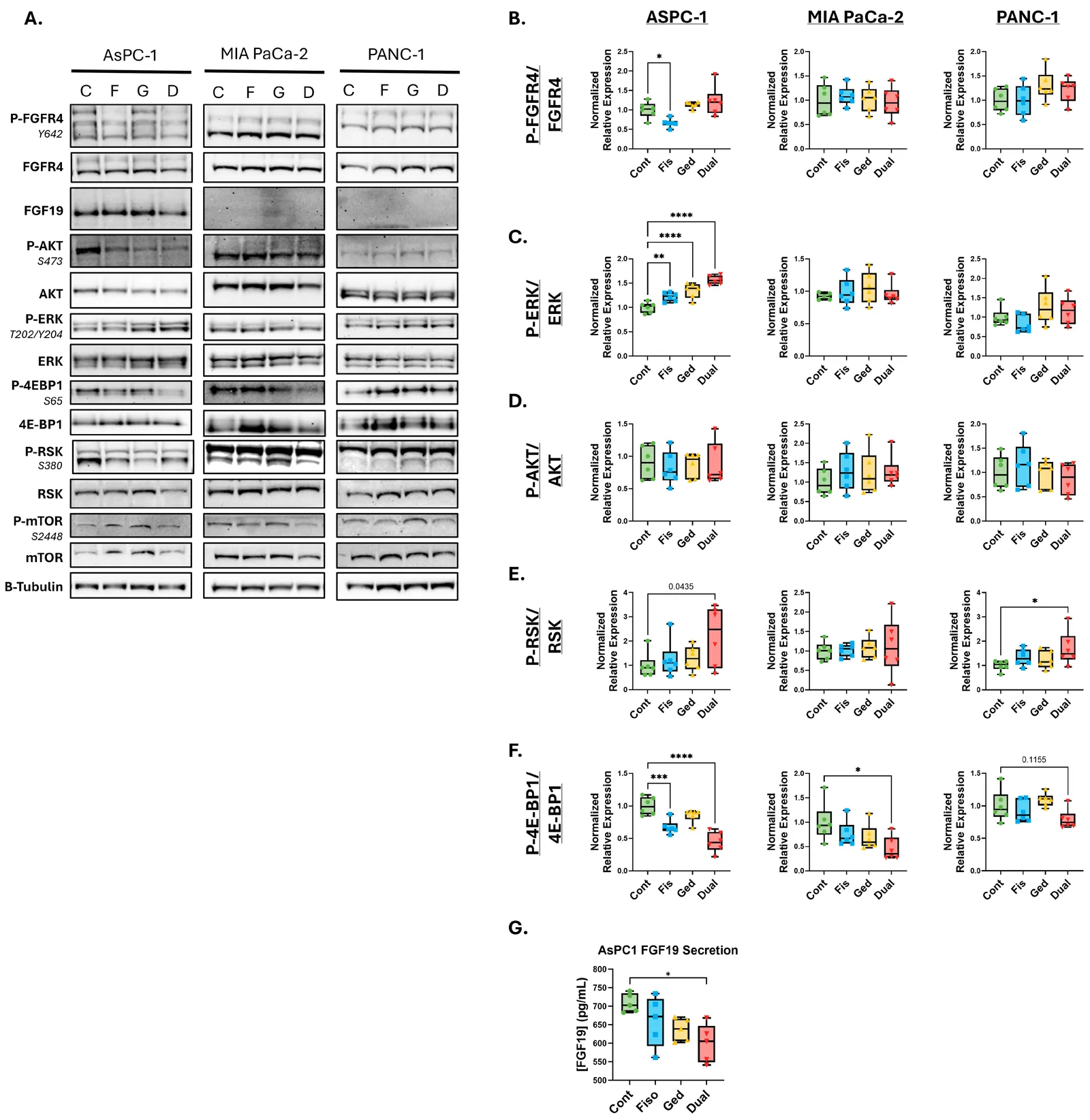
Combined FGFR4 and PI3K-mTOR inhibition modulates MAPK signaling, increases 4E-BP1 suppressive activity, and suppresses FGF19 secretion. (**A**) Representative immunoblots of PI3K–AKT–mTOR and FGF19–FGFR4 pathway components following 24 h of treatment with fisogatinib (10 µM), gedatolisib (10 nM), or combination compared to vehicle in each of AsPC-1, MIA PaCa-2, or PANC-1 cell lines, respectively. Densitometric quantification of (**B**) P-FGFR4/FGFR4, (**C**) P-ERK/ERK, (**D**) P-AKT/AKT, (**E**) P-RSK/RSK, and (**F**) P-4E-BP1/4E-BP1 is shown across all replicates (n = 6). Due to the number of treatment replicates, the blots were performed per cell line and loaded at 30 µg total protein. Antibody incubation and detection times for each respective band were constant. Values were normalized to respective beta-tubulin controls and relative to the vehicle control averages. Combination treatment was associated with cell line-specific modulation of ERK and/or RSK activity downstream of MAPK signaling and was associated with increased 4E-BP1 inhibitory activity that was significant in responsive models. (**G**) ELISA quantification of secreted FGF19 in conditioned media following 24 h treatment of AsPC-1 (n = 5). Combined FGFR4 and PI3K-mTOR inhibition reduced FGF19 secretion in AsPC-1 cells relative to vehicle controls. Data represent mean ± SD. Significance was calculated using one-way ANOVA with multiple comparisons. Statistics: * *p* < 0.05, ** *p* < 0.01, *** *p* < 0.001, **** *p* < 0.0001.

To further characterize signaling responses associated with FGFR4 and PI3K-mTOR co-targeting, FGFR4, PI3K-mTOR, MAPK, and translational signaling components were evaluated by immunoblotting following 24 h of treatment with fisogatinib, gedatolisib, or the combination (Figure 4A, Figures S2B and S3A,B). In AsPC-1 cells, fisogatinib significantly reduced P-FGFR4/FGFR4, confirming on-target inhibition of FGFR4 signaling. Despite this, all treatment groups demonstrated increased P-ERK/ERK relative to vehicle, and combination treatment also increased downstream P-RSK/RSK, thereby suggesting a compensatory activation of MAPK signaling. Irrespective of increased ERK and RSK activity, P-4E-BP1 levels were significantly reduced following fisogatinib and combination treatment. In the MIA PaCa-2 model, combination treatment significantly decreased the P-4E-BP1/4E-BP1 ratio, whereas changes in other signaling components were less pronounced. In PANC-1 cells, combination treatment significantly increased P-RSK/RSK and was associated with a trend toward reduced P-4E-BP1/4E-BP1 levels. Collectively, signaling responses varied across PDAC models, particularly with respect to ERK and RSK activation. Nevertheless, combination treatment was consistently associated with modulation of signaling pathways linked to translational regulation, such as increased 4E-BP1 inhibitory activity in responsive models. Notably, increased ERK-RSK signaling did not correspond with restoration of 4E-BP1 phosphorylation across the PDAC models examined.

### 2.8. Combined FGFR4 and PI3K-mTOR Inhibition Reduces FGF19 Secretion in AsPC-1 Cells

To evaluate the effects of pathway inhibition on ligand availability, cells were plated at equivalent cell numbers and secreted FGF19 levels were quantified in conditioned media following 24 h of treatment relative to a standard curve, per manufacturer’s protocol (Figure 4G). ELISA analysis revealed that MIA PaCa-2 and PANC-1 cells secreted FGF19 at concentrations below the detection limit (10 pg/mL) under all conditions, consistent with their comparably low baseline FGF19-FGFR4 axis activity relative to AsPC-1 cells. In contrast, AsPC-1 cells secreted readily detectable FGF19 and were useful for evaluating associated treatment changes. In AsPC-1, combined fisogatinib and gedatolisib treatment significantly reduced secreted FGF19 compared to vehicle controls. Neither fisogatinib nor gedatolisib monotherapy significantly altered FGF19 levels. These findings indicate that suppression of FGF19 secretion was most pronounced following combined inhibition of FGFR4 and PI3K-mTOR signaling. Whether low FGF19 availability directly contributes to the differential MAPK responses observed across PDAC models remains to be determined. Future studies examining the effects of exogenous FGF19 supplementation in FGF19-low cell lines will help establish whether ligand availability is sufficient to modulate ERK/RSK activation or whether these responses are primarily dictated by intrinsic differences in downstream signaling.

## 3. Discussion

This study supports the concept that dual inhibition of FGFR4 and PI3K–mTOR signaling produces coordinated, model-dependent anti-tumor effects in PDAC and highlights translational control as a shared downstream regulatory node linking oncogenic signaling to the metabolic and biosynthetic demands of PDAC cells. Across PDAC models, combined targeting of FGFR4 and PI3K–mTOR signaling reduced proliferation, clonogenic survival, and migration, and promoted G1 cell-cycle accumulation, whereas apoptotic responses were associated primarily with fisogatinib-containing treatment groups (Figure 1D,E, Figure 2 and Figure 3). These effects were accompanied by modulation of downstream signaling pathways and attenuation of adaptive signaling responses observed with monotherapy, consistent with prior evidence that FGFR4 inhibition alone can elicit variable or paradoxical signaling outcomes in PDAC [8].

At the clinical level, integrative analysis of TCGA-PAAD data provided exploratory support for the relationship between FGFR4 and PI3K-mTOR signaling in PDAC. Spearman correlation analysis demonstrated a statistically significant but modest association between FGFR4 and PI3K–mTOR axis activity (ρ = 0.178), indicating limited shared variance between the two programs at the transcript level. A composite score integrating both pathways separated patients into groups with numerically distinct overall survival (Figure 1A), although the omnibus four-group comparison did not reach significance (*p* = 0.129) and the single nominally significant pairwise contrast was observed in the FGFR4-low/PI3K–mTOR-high group. Pairwise survival comparisons were exploratory in nature and were not adjusted for multiple testing; these findings should therefore be considered hypothesis-generating rather than prognostically confirmatory. Time-dependent ROC analysis showed discriminatory performance near chance at 1 year (AUC = 0.52), with improved but still exploratory performance at 3 and 5 years (Figure 1B). Given the modest cohort size (n = 182), limited patient survival at later time points, and absence of an independent validation cohort, this analysis should be considered hypothesis-generating rather than prognostically confirmatory. These findings are consistent with prior reports describing heterogeneous and sometimes discordant associations between FGFR4 expression and PDAC outcomes [8,10,33]. As our axis scores were derived from transcript-level expression, they represent a simplified proxy for pathway activity and do not capture post-translational regulation, which is a major determinant of PI3K-mTOR signaling in particular. Collectively, these data support a hypothesis-generating relationship between FGFR4 and PI3K–mTOR signaling activity in PDAC and suggest coordinated pathway behavior rather than dominance of either signaling axis alone.

Functionally, PDAC cell lines exhibited distinct pathway dependencies that influenced therapeutic response. Inclusion of FGFR4-high AsPC-1 cells alongside the FGFR4-low models (MIA PaCa-2 and PANC-1) enabled evaluation of whether combined FGFR4 and PI3K-mTOR inhibition retains activity across heterogeneous PDAC backgrounds. AsPC-1 cells, characterized by elevated FGF19 and FGFR4 activity, were generally more responsive to FGFR4 inhibition, consistent with ligand-associated pathway signaling [33]. In contrast, PANC-1 cells showed limited sensitivity to fisogatinib alone but exhibited reduced migration and altered cell-cycle progression following combined pathway targeting. MIA PaCa-2 cells displayed an intermediate response profile (Figure 1C–E). Despite these differences, dual inhibition consistently suppressed tumor-associated phenotypes across models. Although fisogatinib monotherapy produced substantial effects in select assays, particularly in FGFR4-responsive models, combined treatment generally resulted in broader and more consistent suppression of tumor-associated phenotypes while modulating multiple downstream signaling pathways implicated in adaptive responses. ZIP analysis indicated that these effects were primarily additive rather than strongly synergistic (Figure S1B). Nevertheless, the findings support dual pathway targeting as an approach for addressing heterogeneous signaling states and limiting adaptive pathway responses that may reduce the durability of monotherapy.

Mechanistically, dual FGFR4 and PI3K-mTOR inhibition resulted in distinct cell-context-dependent signaling responses across PDAC models (Figure 4). In AsPC-1 cells, fisogatinib reduced P-FGFR4, confirming on-target inhibition in a ligand-dependent context. Across models, one of the most consistent downstream effects was modulation of 4E-BP1, a critical regulator of cap-dependent protein translation and cellular biosynthetic activity. Significant reductions in P-4E-BP1 were observed in AsPC-1 and MIA PaCa-2 cells, with a similar decreasing trend in PANC-1 cells following combination treatment. These findings suggest that modulation of translational regulation may represent a shared downstream response despite differences in baseline signaling activity across PDAC models. An important observation was the emergence of adaptive MAPK activation. AsPC-1 cells exhibited increased ERK phosphorylation following combination treatment and also increased RSK activation. Similarly, PANC-1 cells demonstrated increased RSK activation in response to combination treatment. However, despite increased ERK-RSK signaling, tumor-associated phenotypes remained suppressed, and increased 4E-BP1 inhibitory activity was evident in responsive models. These findings suggest that adaptive MAPK activation alone was insufficient to fully restore downstream 4E-BP1 phosphorylation necessary to sustain tumor growth and survival. Collectively, the results are consistent with the possibility that altered translational regulation contributes to the observed suppression of proliferation and clonogenic growth across models.

In addition to intracellular signaling effects, combined pathway inhibition was associated with altered ligand availability. Dual FGFR4 and PI3K–mTOR inhibition reduced FGF19 secretion in AsPC-1 cells, whereas monotherapies had minimal impact (Figure 4G). This suggests that combined targeting may disrupt autocrine reinforcement of FGFR4 signaling in ligand-dependent contexts. The absence of detectable FGF19 secretion in MIA PaCa-2 and PANC-1 cells further underscores the heterogeneity of FGFR4-axis activity across PDAC models.

Cell context-dependent signaling responses were reflected in coordinated phenotypic outcomes. Combined inhibition promoted G1 accumulation across all PDAC models, whereas apoptotic responses were most evident in fisogatinib-containing treatment groups (Figure 3A–C). Notably, AsPC-1 cells exhibited a more modest apoptotic response than MIA PaCa-2 or PANC-1 despite their high baseline FGFR4 pathway activity, indicating that the magnitude of apoptosis induction did not directly correspond to FGFR4 signaling activity. This may reflect that apoptotic sensitivity depends more on each line’s reliance on the shared downstream translational node than on baseline FGFR4 pathway strength, consistent with our broader model, or possible off-target effects that were not captured in this study. Cell death occurred predominantly through apoptosis rather than necrosis, indicating that growth suppression was associated with a regulated cell death response rather than nonspecific cytotoxicity.

This study has several limitations. Our conclusions regarding FGFR4 and PI3K-mTOR pathway involvement rest on pharmacologic inhibition and correlative signaling data; we did not perform genetic loss-of-function studies (e.g., FGFR4 knockdown or knockout) to confirm target dependence, nor did we evaluate these findings in an in vivo or patient-derived xenograft model. As such, on-target specificity and the in vivo relevance of the observed effects remain to be established. Specifically, reduced P-FGFR4/FGFR4 confirming on-target engagement was demonstrated only in the FGFR4-high AsPC-1 line (Figure 4A,B). Additionally, 4E-BP1 phosphorylation was used as a proxy marker of translational regulation; we did not perform a direct functional assay of cap-dependent translation output (e.g., puromycin incorporation or polysome profiling), and this remains an important direction for future work. Similarly, while the migration assays incorporated mitomycin C to isolate proliferation-independent effects, and the persistence of the combination effect under mitomycin C supports a genuine migration-associated component, contributions from altered cell survival, adhesion, or serum-starvation-induced signaling changes cannot be fully excluded.

Together, these findings support a model in which pharmacologic targeting of FGFR4 and PI3K-mTOR-associated signaling influences a partially interdependent network in PDAC that converges on translational control. In this framework, FGFR4 functions as an upstream regulator of receptor-driven signaling, while mTOR integrates downstream outputs governing protein synthesis and metabolic activity. Inhibition of either pathway alone permits compensatory signaling through parallel or downstream nodes, whereas dual inhibition is associated with modulation of translational regulation and reduced adaptive reprogramming.

Clinically, the pharmacologic agents used in this study have established translational relevance. Fisogatinib (BLU-554), a selective FGFR4 inhibitor, has demonstrated safety and target engagement in early-phase clinical studies of FGF19-positive hepatocellular carcinoma [13]. Gedatolisib (PF-05212384), a dual PI3K-mTORC1/2 inhibitor, has been evaluated across multiple solid tumor settings and continues to advance in clinical development, including combination-based strategies [29,35]. Fisogatinib’s nanomolar potency has been established primarily in FGF19-driven hepatocellular carcinoma, where FGFR4 expression and pathway dependence are highest. In tumor contexts with lower FGFR4-axis dependence, published work has used fisogatinib in the low-to-mid micromolar range to achieve measurable biological effects, including both gastric and ovarian cancer [36,37]. The 10 µM concentration used in the present study was selected based on dose–response data (Figure 1E) as the lowest concentration producing consistent, measurable pathway modulation across the PDAC panel while preserving cell viability for downstream assays. The selection of gedatolisib over earlier-generation dual inhibitors was supported by its ability to more comprehensively suppress feedback reactivation of PI3K–AKT signaling, providing a clinically relevant backbone for combination evaluation.

These findings reinforce that FGFR4 dependence in PDAC is heterogeneous rather than universal, and that its therapeutic relevance is best interpreted alongside PI3K–mTOR pathway status rather than in isolation. These findings provide preliminary support for a biomarker-guided approach, in which combined FGFR4/PI3K–mTOR pathway activity, rather than FGFR4 expression alone, may better identify PDAC tumors most likely to benefit from this combination strategy.

In conclusion, dual targeting of FGFR4 and PI3K–mTOR signaling modulates complementary oncogenic pathways that converge on a marker associated with translational control in PDAC. Reduced 4E-BP1 phosphorylation, together with broad suppression of proliferation, clonogenic survival, migration, and cell cycle progression, supports modulation of this translational marker as an important mechanistic feature of this therapeutic strategy, pending direct functional confirmation of translational output. Notably, apoptotic induction was attributable principally to fisogatinib itself, whereas the combination’s added value over fisogatinib alone was most evident in migration, cell cycle control, and translational suppression. These effects persisted despite evidence of adaptive ERK-RSK activation, indicating that compensatory MAPK signaling alone is insufficient to restore the critical downstream programs required for tumor maintenance. Together, these results provide a preliminary, hypothesis-generating rationale for stratifying PDAC patients based on combined FGFR4/PI3K–mTOR pathway status and for pursuing biomarker-guided combination strategies targeting these pathways in PDAC. Furthermore, the observed activity of dual inhibition in FGFR4-low contexts supports the concept that therapeutic vulnerability is not restricted to FGFR4-addicted tumors but extends across heterogeneous PDAC signaling states. These findings warrant validation in vivo and patient-derived models to further evaluate the therapeutic potential and biomarker applicability of this strategy in PDAC.

## 4. Materials and Methods

### 4.1. Chemical Reagents

Stock solutions for fisogatinib (MedChemExpress, Monmouth Junction, NJ, USA, cat. #HY-100492), gedatolisib (MedChemExpress, cat. #HY-10681), everolimus (MedChemExpress, cat. #HY-10218), 4EGI-1 (MedChemExpress, cat. #HY-19831), omipalisib (MedChemExpress, cat. #HY-10297), and ridaforolimus (MedChemExpress, cat. #HY-50908) were prepared in 100% DMSO and stored at −80 °C. Control groups for all studies used vehicle equating to maximal DMSO concentration across groups (0.11% DMSO for all fisogatinib and gedatolisib studies), and all treatment groups had DMSO concentration adjusted to match this as well. CellTiter 96 Aqueous MTS reagent (Promega, Madison, WI, USA, cat. #G5421) was prepared with Phenazine Methyl Sulfate (TCI America, Portland, OR, USA, cat. #P00835G) according to manufacturer instructions. Mitomycin C (Fisher Scientific, Waltham, MA, USA, cat. #BP25312) was stored at −20 °C at 50 µg/mL.

### 4.2. Cell Culture

Human pancreatic adenocarcinoma cells AsPC-1 (ATCC, Manassas, VA, USA, cat. #CRL-1682, RRID: CVCL_0152), MIA PaCa-2 (ATCC, cat. #CRM-CRL-1420, RRID: CVCL_0428), and PANC-1 (ATCC, cat. #CRL-1469, RRID: CVCL_0480) were from the American Type Culture Collection (ATCC) and L3.6pl was a gift from Dr. Isaiah Fidler (MD Anderson Cancer Center, Houston, TX, USA). Cells were maintained in high-glucose Dulbecco’s Modified Eagle’s Medium (DMEM) (Corning Incorporated, Corning, NY, USA, cat. #MT10013CV0). Growth media contained 10% fetal bovine serum (Sigma, St. Louis, MO, USA, cat. #F2442) and was supplemented with 100 µg/mL penicillin-streptomycin (Corning Incorporated, cat. #30-002-CI). Repeated testing for Mycoplasma contamination was performed using a Mycoplasma Detection Kit (SouthernBiotech, Birmingham, AL, USA, cat. #13100-01), and cell line authentication was performed by ATCC-verified STR profiling.

### 4.3. Survival Modeling of TCGA-PAAD Transcriptomic Data

RNA-sequencing and clinical data for TCGA-PAAD were obtained from The Cancer Genome Atlas via the Genomic Data Commons using TCGAbiolinks (version 2.38.0). STAR-aligned raw count data were downloaded and processed in R (version 4.5.3). Gene-level annotation was performed using TCGA metadata, and duplicate gene identifiers mapping to the same gene symbol were collapsed by summing expression values. Genes lacking valid annotations were excluded.

Two predefined signaling axes were constructed based on canonical pathway components: the FGFR4 axis (FGFR4, FGF19) and the PI3K–mTOR axis (PIK3CA, PDK1, MTOR). Axis scores were calculated as the mean expression of constituent genes and subsequently Z-score normalized across samples. TCGA expression data were matched to clinical metadata using patient barcodes.

Patients were stratified into four groups based on a median split of each axis independently: Low FGFR4/Low PI3K–mTOR, Low FGFR4/High PI3K–mTOR, High FGFR4/Low PI3K–mTOR, and High FGFR4/High PI3K–mTOR. Overall survival (OS) was defined as time from diagnosis to death or last follow-up, with vital status defining event occurrence.

Spearman correlation analysis was performed to assess the association between FGFR4 and PI3K–mTOR axis activity. Kaplan–Meier survival analysis was used to compare overall survival across the four axis-defined groups, with the overall four-group comparison assessed by log-rank test. Pairwise log-rank comparisons were subsequently performed relative to the Low FGFR4/Low PI3K–mTOR reference group.

Time-dependent receiver operating characteristic (ROC) analysis (1, 3, and 5 years) was performed using the timeROC package to evaluate the predictive performance of the combined FGFR4/PI3K–mTOR axis model. All analyses were performed in R, and statistical significance was defined as *p* < 0.05.

### 4.4. MTS Assay

A total of 2500 (MIA PaCa-2) or 5000 (AsPC-1, PANC-1) cells per well were adhered overnight to 96-well plates. Cells were synchronized in serum-free supplemented media for 24 h, and then treated with fisogatinib and/or gedatolisib for 72 h. Subsequently, 20 µL of MTS reagent was added to each well and incubated for 3 h at 37 °C, 5% CO_2_. Absorbance was recorded at 490 nm using an Epoch microplate spectrophotometer (BioTek, Winooski, VT, USA).

### 4.5. Clonogenic Survival Assay

AsPC-1 (1000 cells/well), MIA PaCa-2 (500 cells/well) and PANC-1 (1000 cells/well) were adhered overnight to 12-well plates. Cells were synchronized in serum-free supplemented media for 24 h and then challenged with individual drugs alone or combination in DMEM supplemented with 2% FBS for 9 days with media changes at day 3 and 6. The colonies were stained with 0.5% crystal violet in 20% methanol for 10 min. Excess stain was removed, and the colonies were allowed to air-dry. Wells were imaged using the Incucyte SX5 Live Cell Analysis instrument (Sartorius, Göttingen, Germany). The number of colonies and colony size were evaluated using the ImageJ ColonyArea plugin [38].

### 4.6. Scratch-Wound Assay

AsPC-1, MIA PaCa-2, and PANC-1 cells were seeded at 40,000 cells/well in replicates of 8 per treatment group in 96-well plates. After overnight adherence, cells were incubated for 24 h with serum-free supplemented media to allow a confluent monolayer to be formed for scratch wounding. Cells were treated with mitomycin C or vehicle at 10 µg/mL for 2 h at 37 °C before using the Incucyte 96-well Woundermaker (Sartorius). Cells were treated for 24 h with the inhibitors in serum-free media. Live-cell imaging was conducted on the Incucyte SX5 Live Cell Analysis instrument (Sartorius) for 72 h and analyzed using the scratch wound analysis module.

### 4.7. Apoptosis Assay by Annexin V and 7-AAD

Cells were seeded at 100,000 cells/well in replicates of 6 per treatment group in 24-well plates. After overnight adherence, cells were serum-starved for 24 h and then treated with inhibitors. After treatment, cells were lifted and stained with Annexin V-Pacific Blue (BioLegend, San Diego, CA, USA, cat. #640918) and 7-AAD (BioLegend, cat. #420404) for 20 min at RT. Cells were washed and flow cytometry was performed using a CytoFlex LX (Beckman Coulter, Brea, CA, USA) flow cytometer. Data was analyzed via OMIQ software (version 2026.3) (Dotmatics, Boston, MA, USA).

### 4.8. Cell Cycle Analysis

Cells were seeded in the same manner as for the apoptosis assay. After treatment, cells were lifted, fixed with 70% ethanol for 20 min at RT, and stained with FxCycle™ PI/RNase (Invitrogen, Carlsbad, CA, USA, cat. #F10797) according to manufacturer instructions. Flow cytometry used a CytoFlex LX (Beckman Coulter) flow cytometer, and data was analyzed via OMIQ software (Dotmatics).

### 4.9. Immunoblotting

Cells were seeded at 4.5 × 10^5^ cells/well in replicates of 6 per treatment group in 6-well plates. After overnight adherence, cells were serum-starved for 24 h, then treated with inhibitors in serum-free supplemented media for 24 h. Cells were lysed in RIPA buffer (150 mM NaCl, 1.0% NP-40, 0.5% sodium deoxycholate, 0.1% SDS, 50 mM Tris-Cl; pH 8.0) supplemented with Halt Protease and Phosphatase inhibitor cocktail (Thermo Scientific, Waltham, MA, USA, cat. #78440). Protein concentration was determined using detergent-compatible Bradford assay (Thermo Scientific, cat. #23246). Cleared lysates were loaded on 4–12% Bis-Tris gels (Invitrogen, cat. #NP0321BOX). Protein was transferred to nitrocellulose membranes with Bis-tris transfer buffer (25 mM Bicine, 25 mM Bis-tris, 1.03 mM EDTA free acid). Membranes were blocked with 5% BSA in TBS-T and then incubated overnight at 4 °C with primary antibodies at a 1:1000 dilution for anti-4E-BP1 (CST, Danvers, MA, USA, cat. #9644, RRID:AB_2097841), anti-Beta Tubulin (CST, cat. #2128, RRID:AB_823664), anti-FGF19 (CST, cat. #83348, RRID:AB_2800014), anti-P-4E-BP1 (CST, cat. #9451, RRID:AB_330947), anti-P-AKT Ser473 (CST, cat. #4060, RRID:AB_2315049), anti-P-ERK 1/2 Thr202/Tyr204 (CST, cat. #9101, RRID:AB_331646), anti-P-FGFR4 Tyr642 (Abcam, Cambridge, UK, cat. #ab192589, RRID:AB_3095628), anti-P-mTOR Ser2448 (CST, cat. #2971, RRID:AB_330970), anti-P-p90 RSK Ser380 (CST, cat. #11989, RRID:AB_2687613), anti-AKT (CST, cat. #9272, RRID:AB_329827), anti-ERK 1/2 (CST, cat. #9102, RRID:AB_330744), anti-FGFR4 (Santa Cruz, Dallas, TX, USA, cat. #sc-136988, RRID:AB_2103663), anti-mTOR (CST, cat. #2972, RRID:AB_330978), and anti-p90 RSK1/2/3 (CST, cat. #9355, RRID:AB_659900). Secondary antibodies were goat anti-rabbit peroxidase-conjugated AffiniPure IgG (Jackson ImmunoResearch Laboratories, West Grove, PA, USA, cat. #111-035-003, RRID:AB_2313567) or goat anti-mouse peroxidase-conjugated AffiniPure IgG (Jackson ImmunoResearch Laboratories, cat. #115-035-003, RRID:AB_10015289), both diluted 1:10,000 and used for 1 h at RT. Detection was performed with Pierce ECL Western Blotting Substrate (Thermo Scientific, cat. #32109). A ChemiDoc imaging system (BioRad, Hercules, CA, USA) and FIJI Software (version 1.54t) were used for analysis with equal exposure times for each unique protein.

### 4.10. ELISA

After drug treatment for immunoblotting, conditioned media were collected for use in an ELISA. A sandwich ELISA was then performed on aliquots of the diluted conditioned media, using the Human FGF19 DuoSet ELISA (R&D Systems, Minneapolis, MN, USA, cat. #DY969) paired with the DuoSet ELISA Ancillary Reagent Kit 2 (R&D Systems, cat. #DY008), according to manufacturer’s protocol.

### 4.11. Statistics

All results were analyzed using GraphPad Prism version 11.0.0 with the appropriate statistical test. * = *p* < 0.05, ** = *p* < 0.01, *** = *p* < 0.001, **** = *p* < 0.0001.

## 5. Conclusions

This study provides pharmacologic and correlative evidence for a functional convergence between FGFR4 and PI3K–mTOR signaling in pancreatic ductal adenocarcinoma, with reduced 4E-BP1 phosphorylation, a marker associated with translational control, emerging as a shared downstream readout despite heterogeneous upstream pathway dependence across models. Dual inhibition consistently suppressed proliferation, clonogenic survival, migration, and cell cycle progression, with combination treatment providing its clearest added benefit over FGFR4 inhibition alone in these domains rather than in apoptosis induction, which was driven principally by fisogatinib across all three cell lines tested. This distinction indicates that the combination’s therapeutic value lies primarily in its ability to constrain tumor-associated phenotypes and translational output more broadly and consistently, rather than in potentiating cell death beyond what FGFR4 blockade achieves on its own.

Compensatory MAPK activation was observed following combination treatment in select models, yet this adaptive response did not restore 4E-BP1 phosphorylation or reverse the suppression of tumor-associated phenotypes, supporting translational control as a durable node of vulnerability that is not readily circumvented by parallel pathway reactivation. Together with the transcriptomic evidence linking combined FGFR4/PI3K–mTOR pathway activity to reduced overall survival in the TCGA-PAAD cohort, these findings provide preliminary, hypothesis-generating support for a biomarker-guided approach in which combined pathway status, rather than FGFR4 expression alone, may better identify PDAC tumors likely to benefit from this combination strategy. These results warrant further validation in vivo and in patient-derived models to define the therapeutic potential and biomarker applicability of FGFR4/PI3K–mTOR co-targeting in pancreatic cancer.

## Data Availability

RNA-sequencing and clinical data for TCGA-PAAD were obtained from The Cancer Genome Atlas via the Genomic Data Commons using TCGAbiolinks. STAR-aligned raw count data were downloaded and processed in R (version 4.5.3). The original contributions presented in this study are included in the article/Supplementary Materials. Further inquiries can be directed to the corresponding author.

## Supplementary Materials

The following supporting information can be downloaded at: https://www.mdpi.com/article/10.3390/ijms27177722/s1.

## Abbreviations

The following abbreviations are used in this manuscript:

4E-BP1	Eukaryotic Translation Initiation Factor 4E-Binding Protein 1
4EGI-1	4E–eIF4G Interaction Inhibitor-1
7-AAD	7-Aminoactinomycin D
AKT	Protein Kinase B
ATCC	American Type Culture Collection
AUC	Area Under the Curve
BSA	Bovine Serum Albumin
CST	Cell Signaling Technology
DMEM	Dulbecco’s Modified Eagle’s Medium
DMSO	Dimethyl Sulfoxide
ECL	Enhanced Chemiluminescence
EDTA	Ethylenediaminetetraacetic Acid
ELISA	Enzyme-Linked Immunosorbent Assay
ERK	Extracellular Signal-Regulated Kinase
FGF19	Fibroblast Growth Factor 19
FGFR4	Fibroblast Growth Factor Receptor 4
G1 Phase	Gap 1 Phase
G2/M Phase	Gap 2/Mitosis Phase
IRS1	Insulin Receptor Substrate 1
KRAS	Kirsten Rat Sarcoma Viral Oncogene Homolog
MAPK	Mitogen-Activated Protein Kinase
MTS	3-(4,5-Dimethylthiazol-2-yl)-5-(3-carboxymethoxyphenyl)-2-(4-sulfophenyl)-2H-tetrazolium
mTOR	Mechanistic Target of Rapamycin
mTORC1	Mechanistic Target of Rapamycin Complex 1
mTORC2	Mechanistic Target of Rapamycin Complex 2
NP-40	Nonidet P-40
OS	Overall Survival
PAAD	Pancreatic Adenocarcinoma
PDAC	Pancreatic Ductal Adenocarcinoma
PDK1	3-Phosphoinositide-Dependent Protein Kinase-1
PI3K	Phosphoinositide 3-Kinase
PIK3CA	Phosphatidylinositol-4,5-Bisphosphate 3-Kinase Catalytic Subunit Alpha
RIPA	Radioimmunoprecipitation Assay
ROC	Receiver Operating Characteristic
RSK	Ribosomal S6 Kinase
SDS	Sodium Dodecyl Sulfate
S Phase	Synthesis Phase
STAR	Spliced Transcripts Alignment to a Reference
TCGA	The Cancer Genome Atlas
TBS-T	Tris-Buffered Saline with Tween 20
ZIP	Zero Interaction Potency

## References

[1] Siegel R.L., Kratzer T.B., Giaquinto A.N., Sung H., Jemal A. (2025). Cancer statistics, 2025. CA Cancer J. Clin..

[2] Dilly J., Hoffman M.T., Abbassi L., Li Z., Paradiso F., Parent B.D., Hennessey C.J., Jordan A.C., Morgado M., Dasgupta S. (2024). Mechanisms of Resistance to Oncogenic KRAS Inhibition in Pancreatic Cancer. Cancer Discov..

[3] Bear A.S., Vonderheide R.H., O’Hara M.H. (2020). Challenges and Opportunities for Pancreatic Cancer Immunotherapy. Cancer Cell.

[4] Principe D.R., Korc M., Kamath S.D., Munshi H.G., Rana A. (2021). Trials and tribulations of pancreatic cancer immunotherapy. Cancer Lett..

[5] Guo C., Zhou N., Lu Y., Mu M., Li Z., Zhang X., Tu L., Du J., Li X., Huang D. (2024). FGF19/FGFR4 signaling contributes to hepatocellular carcinoma survival and immune escape by regulating IGF2BP1-mediated expression of PD-L1. Biomed. Pharmacother..

[6] Shi S., Zhang Q., Zhang K., Chen W., Xie H., Pan S., Xue Z., You B., Zhao J., You Y. (2024). FGF19 promotes nasopharyngeal carcinoma progression by inducing angiogenesis via inhibiting TRIM21-mediated ANXA2 ubiquitination. Cell. Oncol..

[7] Chen X., Chen J., Feng W., Huang W., Wang G., Sun M., Luo X., Wang Y., Nie Y., Fan D. (2023). FGF19-mediated ELF4 overexpression promotes colorectal cancer metastasis through transactivating FGFR4 and SRC. Theranostics.

[8] D’Agosto S., Pezzini F., Veghini L., Delfino P., Fiorini C., Temgue Tane G.D., Del Curatolo A., Vicentini C., Ferrari G., Pasini D. (2022). Loss of FGFR4 promotes the malignant phenotype of PDAC. Oncogene.

[9] Crawford K.J., Humphrey K.S., Cortes E., Wang J., Kumarasamy V., Wan Y., Long M.D., Feigin M.E., Witkiewicz A.K., Mann K.M. (2025). Targeting FGFR4 abrogates HNF1A-driven metastasis in pancreatic ductal adenocarcinoma. Mol. Cancer.

[10] Braun M., Durślewicz J., Sołek J., Nowicka Z., Zielińska A., Antosik P., Grzanka D. (2025). High FGFR4 protein expression, but not FGFR1 or FGFR2, predicts poor prognosis in pancreatic ductal adenocarcinoma. BMC Cancer.

[11] Gao L., Lang L., Zhao X., Shay C., Shull A.Y., Teng Y. (2019). FGF19 amplification reveals an oncogenic dependency upon autocrine FGF19/FGFR4 signaling in head and neck squamous cell carcinoma. Oncogene.

[12] Li F., Li Z., Han Q., Cheng Y., Ji W., Yang Y., Lu S., Xia W. (2020). Enhanced autocrine FGF19/FGFR4 signaling drives the progression of lung squamous cell carcinoma, which responds to mTOR inhibitor AZD2104. Oncogene.

[13] Kim R.D., Sarker D., Meyer T., Yau T., Macarulla T., Park J.-W., Choo S.P., Hollebecque A., Sung M.W., Lim H.-Y. (2019). First-in-Human Phase I Study of Fisogatinib (BLU-554) Validates Aberrant FGF19 Signaling as a Driver Event in Hepatocellular Carcinoma. Cancer Discov..

[14] Wang S., Zhao D., Tian R., Shi H., Chen X., Liu W., Wei L. (2016). FGF19 Contributes to Tumor Progression in Gastric Cancer by Promoting Migration and Invasion. Oncol. Res..

[15] Xin Z., Song X., Jiang B., Gongsun X., Song L., Qin Q., Wang Q., Shi M., Liu X. (2018). Blocking FGFR4 exerts distinct anti-tumorigenic effects in esophageal squamous cell carcinoma. Thorac. Cancer.

[16] Turkington R.C., Longley D.B., Allen W.L., Stevenson L., McLaughlin K., Dunne P.D., Blayney J.K., Salto-Tellez M., Van Schaeybroeck S., Johnston P.G. (2014). Fibroblast growth factor receptor 4 (FGFR4): A targetable regulator of drug resistance in colorectal cancer. Cell Death Dis..

[17] Sasaki N., Gomi F., Yoshimura H., Yamamoto M., Matsuda Y., Michishita M., Hatakeyama H., Kawano Y., Toyoda M., Korc M. (2020). FGFR4 Inhibitor BLU9931 Attenuates Pancreatic Cancer Cell Proliferation and Invasion While Inducing Senescence: Evidence for Senolytic Therapy Potential in Pancreatic Cancer. Cancers.

[18] Wei F., Zhang Y., Geng L., Zhang P., Wang G., Liu Y. (2015). mTOR inhibition induces EGFR feedback activation in association with its resistance to human pancreatic cancer. Int. J. Mol. Sci..

[19] Hassan Z., Schneeweis C., Wirth M., Veltkamp C., Dantes Z., Feuerecker B., Ceyhan G.O., Knauer S.K., Weichert W., Schmid R.M. (2018). MTOR inhibitor-based combination therapies for pancreatic cancer. Br. J. Cancer.

[20] Rozengurt E., Soares H.P., Sinnet-Smith J. (2014). Suppression of feedback loops mediated by PI3K/mTOR induces multiple overactivation of compensatory pathways: An unintended consequence leading to drug resistance. Mol. Cancer Ther..

[21] Soares H.P., Ming M., Mellon M., Young S.H., Han L., Sinnet-Smith J., Rozengurt E. (2015). Dual PI3K/mTOR Inhibitors Induce Rapid Overactivation of the MEK/ERK Pathway in Human Pancreatic Cancer Cells through Suppression of mTORC2. Mol. Cancer Ther..

[22] Chandarlapaty S., Sawai A., Scaltriti M., Rodrik-Outmezguine V., Grbovic-Huezo O., Serra V., Majumder P.K., Baselga J., Rosen N. (2011). AKT inhibition relieves feedback suppression of receptor tyrosine kinase expression and activity. Cancer Cell.

[23] O’Reilly K.E., Rojo F., She Q.-B., Solit D., Mills G.B., Smith D., Lane H., Hofmann F., Hicklin D.J., Ludwig D.L. (2006). mTOR inhibition induces upstream receptor tyrosine kinase signaling and activates Akt. Cancer Res..

[24] Driscoll D.R., A Karim S., Sano M., Gay D.M., Jacob W., Yu J., Mizukami Y., Gopinathan A., Jodrell D.I., Evans T.J. (2016). mTORC2 Signaling Drives the Development and Progression of Pancreatic Cancer. Cancer Res..

[25] Kong B., Wu W., Cheng T., Schlitter A.M., Qian C., Bruns P., Jian Z., Jäger C., Regel I., Raulefs S. (2016). A subset of metastatic pancreatic ductal adenocarcinomas depends quantitatively on oncogenic Kras/Mek/Erk-induced hyperactive mTOR signalling. Gut.

[26] Zhao M., Scott S., Evans K.W., Yuca E., Saridogan T., Zheng X., Wang H., Korkut A., Pico C.X.C., Demirhan M. (2021). Combining Neratinib with CDK4/6, mTOR, and MEK Inhibitors in Models of HER2-positive Cancer. Clin. Cancer Res..

[27] Belmont P.J., Jiang P., McKee T.D., Xie T., Isaacson J., Baryla N.E., Roper J., Sinnamon M.J., Lee N.V., Kan J.L.C. (2014). Resistance to dual blockade of the kinases PI3K and mTOR in KRAS-mutant colorectal cancer models results in combined sensitivity to inhibition of the receptor tyrosine kinase EGFR. Sci. Signal..

[28] Munster P., Aggarwal R., Hong D., Schellens J.H., Van Der Noll R., Specht J., Witteveen P.O., Werner T.L., Dees E.C., Bergsland E. (2016). First-in-Human Phase I Study of GSK2126458, an Oral Pan-Class I Phosphatidylinositol-3-Kinase Inhibitor, in Patients with Advanced Solid Tumor Malignancies. Clin. Cancer Res..

[29] Wainberg Z.A., Alsina M., Soares H.P., Braña I., Britten C.D., Del Conte G., Ezeh P., Houk B., Kern K.A., Leong S. (2017). A Multi-Arm Phase I Study of the PI3K/mTOR Inhibitors PF-04691502 and Gedatolisib (PF-05212384) plus Irinotecan or the MEK Inhibitor PD-0325901 in Advanced Cancer. Target. Oncol..

[30] Fahy B.N., Schlieman M., Virudachalam S., Bold R.J. (2003). AKT inhibition is associated with chemosensitisation in the pancreatic cancer cell line MIA-PaCa-2. Br. J. Cancer.

[31] Mao T., Zhang X., Xu H., Zhang X., Ge W., Li S., Ma J., Yue M., Xue S., Cui J. (2022). HDACs/mTOR inhibitor synergizes with pyrotinib in HER2-positive pancreatic cancer through degradation of mutant P53. Cancer Cell Int..

[32] Deng X., Hu J., Ewton D.Z., Friedman E. (2014). Mirk/dyrk1B kinase is upregulated following inhibition of mTOR. Carcinogenesis.

[33] Chia L., Wang B., Kim J.-H., Luo L.Z., Shuai S., Herrera I., Chen S.Y., Li L., Xian L., Huso T. (2023). HMGA1 induces FGF19 to drive pancreatic carcinogenesis and stroma formation. J. Clin. Investig..

[34] Bruns C.J., Harbison M.T., Kuniyasu H., Eue I., Fidler I.J. (1999). In vivo selection and characterization of metastatic variants from human pancreatic adenocarcinoma by using orthotopic implantation in nude mice. Neoplasia.

[35] Shapiro G.I., Bell-McGuinn K.M., Molina J.R., Bendell J., Spicer J., Kwak E.L., Pandya S.S., Millham R., Borzillo G., Pierce K.J. (2015). First-in-Human Study of PF-05212384 (PKI-587), a Small-Molecule, Intravenous, Dual Inhibitor of PI3K and mTOR in Patients with Advanced Cancer. Clin. Cancer Res..

[36] Zhang X., Zhang X., Han R., Wang Z., Yang Q., Huang Y., Yan Y. (2022). BLU-554, A selective inhibitor of FGFR4, exhibits anti-tumour activity against gastric cancer in vitro. Biochem. Biophys. Res. Commun..

[37] Zeng R., Chen X., Chen Y., Dong J. (2025). FGFR4 inhibition augments paclitaxel-induced cell death in ovarian cancer. Int. Immunopharmacol..

[38] Guzmán C., Bagga M., Kaur A., Westermarck J., Abankwa D. (2014). ColonyArea: An ImageJ plugin to automatically quantify colony formation in clonogenic assays. PLoS ONE.

