# Supplementary material for: Dual Targeting of FGFR4 and PI3K–mTOR Suppresses Tumor-Associated Phenotypes in Pancreatic Ductal Adenocarcinoma
Source: Int J Mol Sci. 2026 Aug 28;27(17):7722. doi: 10.3390/ijms27177722 (PMC13566174; doi:10.3390/ijms27177722)
Supplement: Supplementary file 1 [file ijms-27-07722-s001.zip › ijms-4450691-supplementary.pdf]

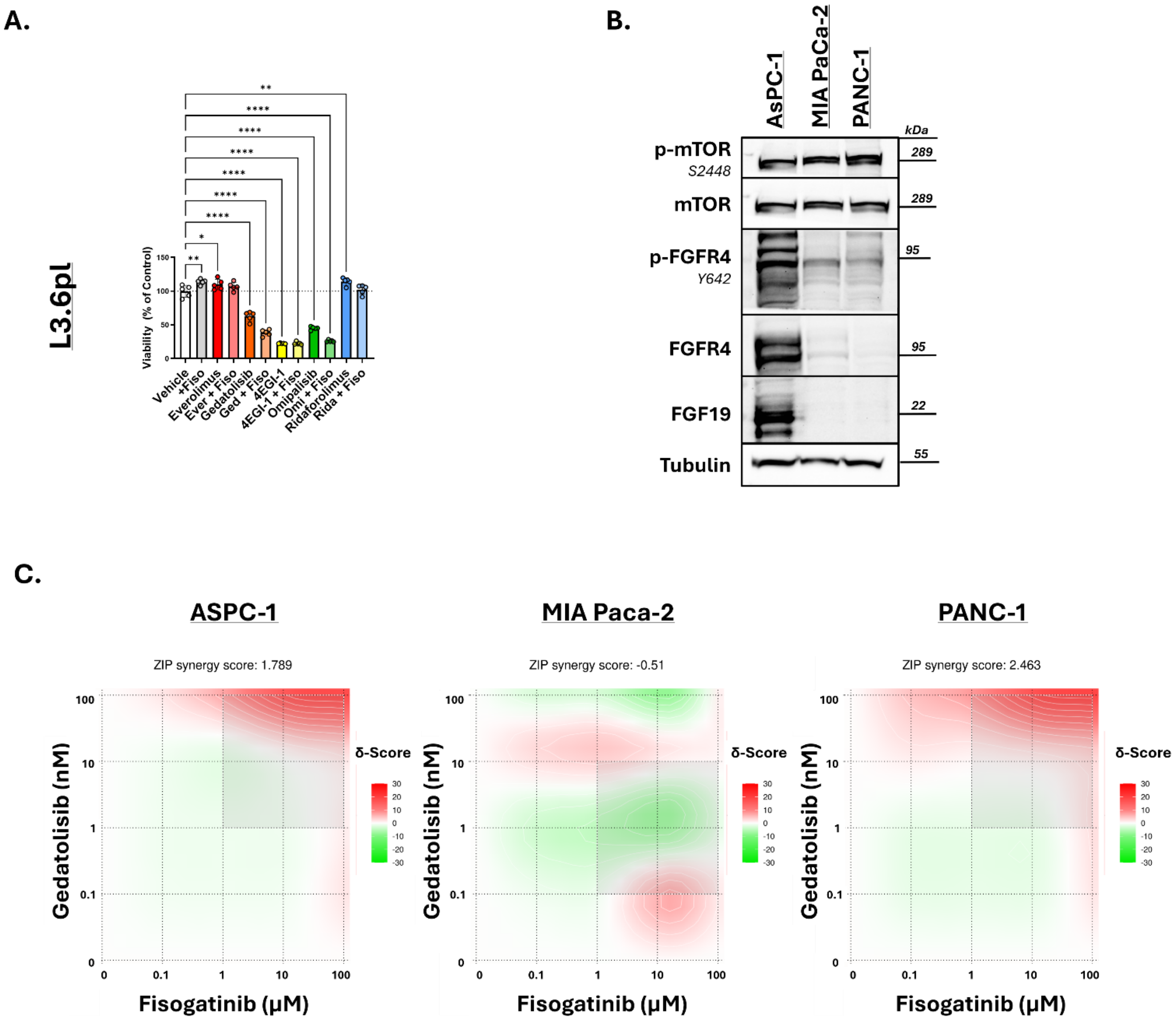

Figure S1. Combination Screening of L3.6pl and ZIP Synergy of Fisogatinib and Gedatolisib of PDAC Cell lines. (A) Cell viability following treatment with mTOR pathway inhibitors (everolimus, 1 µM; gedatolisib, 10 nM; 4EGI-1, 10 µM; omipalisib 10 nM; ridaforolimus, 10 nM) ± fisogatinib (10 µM) for 72 h in L3.6pl. (B) Immunoblot analysis comparing relative baseline expression of FGF19–FGFR4 and mTOR pathway components across cell lines loaded at 50 µg total protein on the same blot. AsPC-1 cells exhibited elevated FGF19–FGFR4 axis activity, whereas MIA PaCa-2 and PANC-1 represented lower FGFR4-axis contexts. mTOR pathway expression was detected across all models. (C) ZIP synergy of AsPC-1, MIA-PaCa-2, and PANC-1 following 72 h treatment with vehicle, fisogatinib (10 µM), gedatolisib (10 nM), or in combination based on viability relative to control determined by MTS assay. ZIP Synergy was then determined through the use of SynergyFinder 2.0 (accessed on 1

June 2026). Significance was calculated using one-way ANOVA with multiple comparisons. Statistics: * $p < 0.05$, ** $p < 0.01$, *** $p < 0.001$, **** $p < 0.0001$.

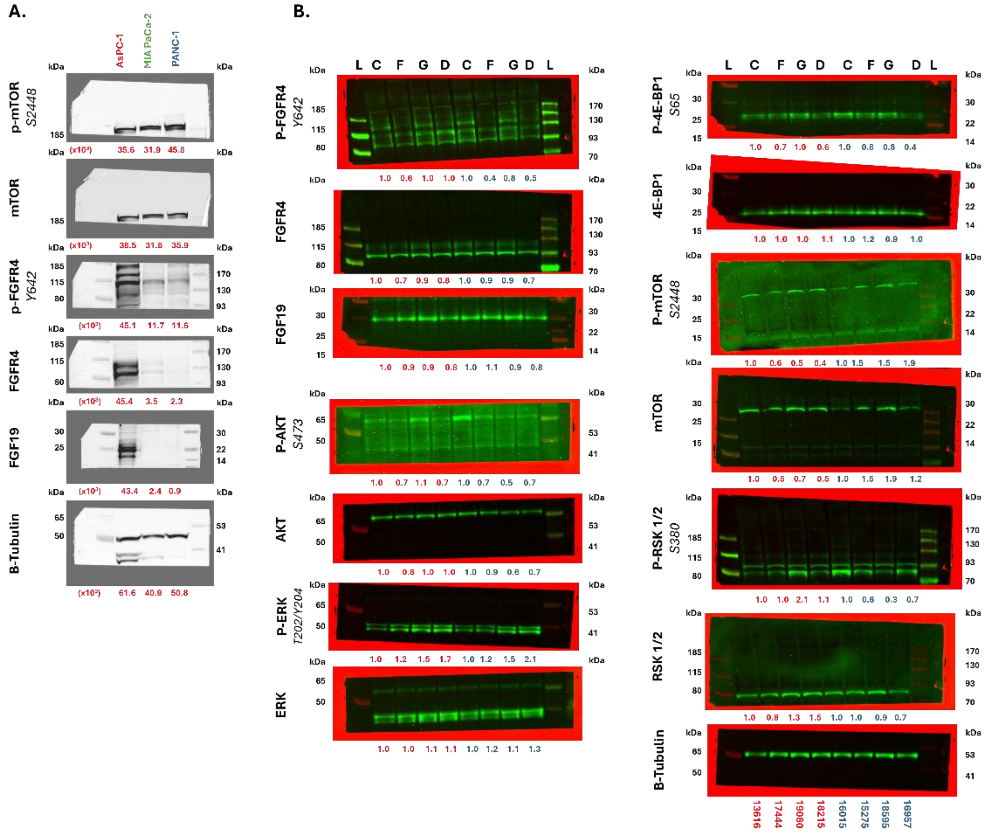

Figure S2. Annotated Whole Immunoblot Sections. Full uncropped composite immunoblots (overlayed colorimetric and chemiluminescent images) corresponding to representative blots shown in A) Figure 1 and B) AsPC-1 replicates shown in Figure 4. A) Immunoblot sections were probed for the indicated proteins in AsPC-1, MIA PaCa-2, or PANC-1 cells following 24 h serum starvation. Values beneath the immunoblots represent absolute band intensities for each corresponding lane (text color; red=left replicate, blue=right replicate). B) Immunoblot sections were probed for the indicated proteins in AsPC-1, MIA PaCa-2, or PANC-1 cells following 24 h serum starvation and treatment with control (C), fisogatinib (F), gedatolisib (G), or dual combination therapy (D). Values beneath each immunoblot represent band intensity ratios normalized to β-tubulin and expressed relative to the corresponding control lane for each cell line. Values beneath the β-tubulin immunoblots represent absolute band intensities. Molecular weight ladders (L) are shown on the left and right sides of each immunoblot.

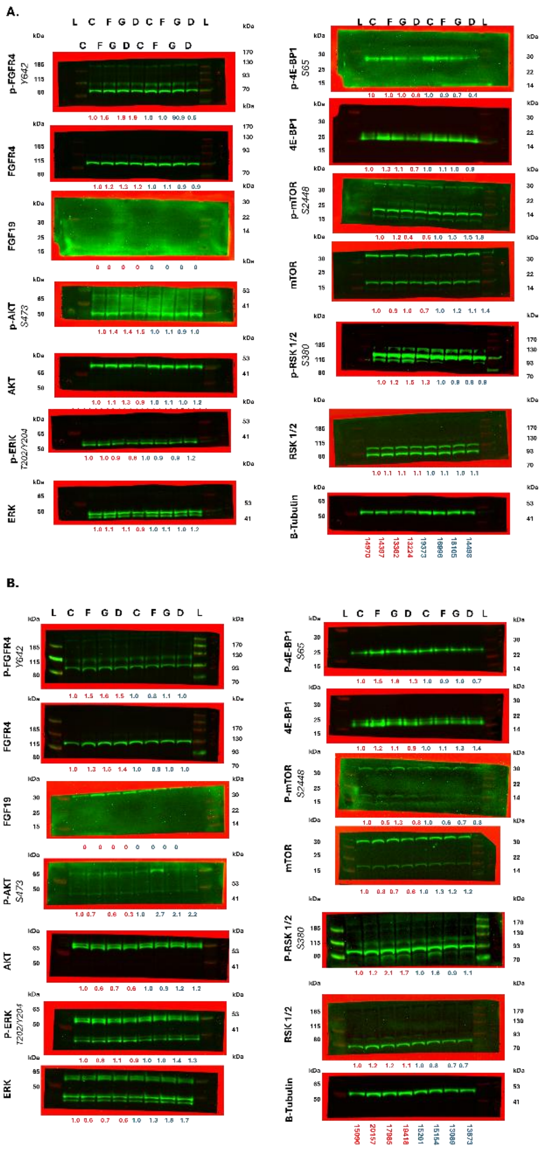

Figure S3. Annotated Whole Immunoblot Sections of MIA-PaCa-2 and PANC-1. Full uncropped composite immunoblots (overlayed colorimetric and chemiluminescent images) corresponding to (A) MIA-PaCa-2 and (B) PANC-1 representative blots shown Figure 4. A) Immunoblot sections were probed for the indicated proteins in MIA PaCa-2, or PANC-1 cells following 24 h serum starvation. Values beneath the immunoblots represent absolute band intensities for each corresponding lane (text color; red=left replicate, blue=right replicate). B) Immunoblot sections were probed for the indicated proteins in MIA PaCa-2 or PANC-1 cells following 24 h serum starvation and treatment with control (C), fisogatinib (F), gedatolisib (G), or dual combination therapy (D). Values beneath each immunoblot represent band intensity ratios normalized to β-tubulin and expressed relative to the corresponding control lane for each cell line. Values beneath the β-tubulin immunoblots represent absolute band intensities. Molecular weight ladders (L) are shown on the left and right sides of each immunoblot.
